# Clinical audit of POM-V / POM prescriptions by remote consultation via a veterinary video telemedicine smartphone application

**DOI:** 10.18849/ve.v7i2.553

**Published:** 2022-06-08

**Authors:** Sheila Smith+, Tasmin Day+, Samantha Webster+, Sam Davies+, Trevor Hardcastle+, Adele Williams+

**Keywords:** STRESS

## Abstract

There is an erratum to this paper published in Veterinary Evidence Vol 7, Issue 2 (2022): 10.18849/VE.V7I2.627

**Objective:**

To assess outcomes of a limited period (7 months) of remote video consultation with prescribing of prescription-only (POM) or prescription-only-veterinary (POM-V) medications by Royal College of Veterinary Surgeons (RCVS) registered veterinary surgeons to UK clients via a veterinary telemedicine smartphone application.

**Background:**

Objective evidence is needed to inform the veterinary profession on the impact that remote prescribing, without physical examination in person, has on animal health and welfare. During the COVID-19 pandemic, the RCVS allowed remote prescribing temporarily.

**Methods:**

Clinical records from all veterinary video consultations from 1 April–31 October 2020 were reviewed. Details were assessed pertaining to: signalment, body system / disease categories managed, referrals into practice, medication classes prescribed and outcomes following POM-V / POM medications. Records of adverse events and antimicrobial prescribing were reviewed.

**Results:**

16.6% (3,541/21,383) of video consults had a POM-V / POM prescribed; with a (mild) adverse event rate of 0.8% (30/3541). Antibacterials were prescribed in 5.88% of all consultations (1,258/21,383), 99.3% (1249/1258) being first line. Follow-up on prescribing was available in 67.7% (2,399/3541) of cases. 89% (2135/2399) of all known treatment outcomes were complete or had an expected response to treatment. Dermatological disease was the most common body system / disease category seen and prescribed for.

**Conclusion:**

Low prescribing rates (including antibacterials) were recorded, treatments were efficacious and no harm was done by prescribing remotely via a veterinary video consult app.

**Custom Section:**

Veterinary surgeons and governing bodies are invited to use the information provided in this clinical audit to inform decisions on the suitability of remote consultations and prescribing in veterinary medicine.

## INTRODUCTION

This manuscript is a clinical audit from a UK-based virtual veterinary telemedicine provider. This audit examines prescribing of prescription-only (POM) and prescription-only-veterinary (POM-V) medications that occurred as a result of veterinary-led video consultations undertaken during a 7 month period (April–October 2020) when remote prescribing without physical examination was permitted under Royal College of Veterinary Surgeons (RCVS) emergency measures due to the COVID-19 pandemic. 

### Background on the smartphone application and remote veterinary telemedicine service provision

The UK-based company producing this clinical audit (Vet-AI) has a smartphone veterinary telemedicine application (Joii Petcare (2021), available on iOS and Android). Dog and cat owners are able to download the app for free on their smartphones, sign up to the app, agree to terms and conditions, and register their pet(s). The functions within the app include options for a client to directly choose to connect to a video consultation with a veterinary surgeon, or to check symptoms via a symptom checker. The symptom checker, designed and developed by veterinary surgeons, directs clients through a series of questions to triage the presenting complaint and direct appropriately depending on the severity of the presenting complaint. If there is an emergency, urgent or serious problem identified, the client is directed to contact a physical veterinary practice, and location services within the app highlight veterinary practices within the local area. If a non-serious / non-urgent problem is identified that still warrants veterinary advice, a veterinary video consultation is suggested. There is the option for owners to have video consultations with registered veterinary nurses if that is the appropriate course of action. There is also the facility for video consultation with a CCAB qualified behaviourist, but only after an initial video consultation with a veterinary surgeon. Veterinary video consultations are available 24/7. Clinicians interact with clients via video link on a bespoke practice management software system where clinical notes are recorded. Clinical records and data are stored in the company’s secure database. The company has a dedicated data protection officer ensuring GDPR compliance and confidentiality of client and pet data (Joii Pet Care, 2021).

All of the veterinary surgeons providing consultations via the app are registered with the RCVS to practice in the UK, and have been qualified and in practice for at least 3 years before joining Vet-AI. All veterinary nurses are qualified and registered with the RCVS. The veterinary surgeons are predominantly part-time and work variable shift patterns. During the period of prescribing, under the temporary RCVS guidance, from April–October 2020 there was no physical practice associated with the company and the clinicians all worked remotely. 

### Follow-up and sharing of clinical information

All clients are provided with an in-app treatment plan at the end of a video consultation. Clinical records are also kept for every consultation, and are provided to a client’s local registered veterinary practice when appropriate.  Clinical record keeping includes the mandatory selection of veterinary nomenclature (VeNOM, 2022) codes to indicate top differential diagnosis / diagnoses at the time of consultation, with the purpose of aiding to identify disease classification types within clinical records.

All clients that had video consultations that resulted in prescription of a POM-V / POM within the app were booked a follow-up veterinary video consult appointment via the app to assess response to treatment and identify any problems arising, or if alternatives needed to be pursued. If clients failed to engage in a follow-up video consultation then they were emailed requesting follow-up information; any follow-up information provided by email was entered into the clinical notes. Clinical history from a patient’s physical veterinary practice was sought (with owner permission) for any animal prescribed a POM-V / POM; chronic medication prescriptions were refused if a full and accurate clinical history could not be obtained. In emergency situations where acute medication was needed to be prescribed for animal welfare reasons, prescribing occurred and a history was sought (or several attempts made to obtain the history) from the registered veterinary practice (following guidance set out in the RCVS code of conduct 5.7). When a patient treated with POM-V / POM remotely was referred into a physical veterinary practice, clinical history was forwarded to that practice (with owner permission).

### Telehealth excellence and clinical monitoring

Vet-AI has an internal telehealth excellence team of clinicians, Joii Scientific Advisory Committee (the authors), dedicated to monitoring clinical practice standards and auditing of clinical data to help produce structured clinical guidelines for the veterinary team to maximise the quality of remote consultation.

They also collaborate evidence from the veterinary and human medical communities to develop best practice telemedicine guidelines. Robust literature reviews govern and underpin all clinical decision-making and guide regular clinical meetings. Best practice and gold standard is the level aspired to, monitored and achieved.

Pharmacovigilance data is collected, assessed and reported to the veterinary medicines directorate (VMD, 2022). The company has a proactive approach to assessing follow-up and adverse event data after all prescriptions.

The team ensures that all clinicians have a structured format for their video consultations and that a full and thorough remote video consultation is performed. 

### Remote prescribing rules in the veterinary profession

The RCVS provide the following information in their Code of Professional Conduct regarding the use of telemedicine (RCVS, 2021):

2.29 Specific advice provided remotely, for example via phone or video-link or without additional physiological data (commonly referred to as telemedicine or telehealth), should only be given to the extent appropriate without a physical examination of the animal. The more specific the advice, the more likely it is that the animal’s owner should be advised to consult a veterinary surgeon in person for a physical examination. In this scenario, the animal owner should be asked to provide the veterinary surgeon carrying out the physical examination with a copy of any advice given remotely.

2.30 Veterinary surgeons should ensure as far as possible that the provision of specific advice provided remotely does not compromise welfare, since the animal has been examined and there is no ability to monitor the animal.

Under normal circumstances, a veterinary surgeon can only prescribe POM-V / POM medications for a pet animal under their care in the UK if they have physically examined the individual animal in person.

### Emergency measures during the COVID-19 pandemic

As an emergency measure during the COVID-19 pandemic, the RCVS released temporary guidance regarding prescription of POM-V / POM medications by veterinary surgeons, due to the extenuating circumstances that emerged with the requirements for en masse public isolation (RCVS, 2020).

This time period provided the ideal opportunity to assess the impact of remote prescribing on animal health and welfare. This can be examined in this clinical audit by detailing the percentage of veterinary video consultations that resulted in POM-V / POM prescriptions, which medications were prescribed, the common body system / disease categories (grouped by the veterinary nomenclature VeNOM codes within the clinical records) that resulted in prescriptions, and what the outcomes were for those treatments where follow-up was available. Recording of any adverse events could be reviewed, as can the use of antimicrobials to examine antimicrobial prescription trends and frequency.

### Remote prescribing company policy

POM-V / POM medications were only prescribed if the following criteria could be met, as per RCVS guidance (RCVS, 2020):

- Enough information could be obtained to assess the pet sufficiently without a hands-on physical exam
- There was no suitable alternative medication of a POM-VPS, NFA-VPS (Non-Food Animal – Veterinarian, Pharmacist, Suitably Qualified Person) or AVM-GSL (Authorised Veterinary Medicine – General Sales List) category
- The benefit to the animal and / or public health risk outweighed the risk
- Immediate / un-delayed action was required in the interests of animal welfare
- The client was fully informed of any risks associated with remote prescribing for the given condition

Detailed notes were made for any decision to remotely prescribe and the reasoning behind it.

Any consultation resulting in medication being prescribed was booked in for a follow-up consultation at the time of recommending the product. The interval between initial and follow-up consultation was determined on a case-by-case basis by the clinician involved.

If the client had not returned for their follow-up appointment, an email was sent to their registered email address. The email made a general enquiry into the state of the pet having been treated, as well as asking the following three questions:

1. Are the symptoms still present?
2. Are you satisfied with the outcome?
3. Has your pet experienced any adverse events in relation to the medication prescribed?

### Evidence around telemedicine and remote prescribing

Vet-to-vet telemedicine has existed since the 1980’s, when telecardiology was utilised to allow general practice veterinarians to seek support from specialist colleagues (Robertson, 1999).

With the advent of digital technologies and live video streaming, veterinarian-to-client telemedicine is a new and emerging service within the veterinary sector globally.

There is controversy within the profession regarding the suitability of this method of vet-pet-client contact for maintaining professional standards and protecting animal health and welfare, given the inability to perform a physical examination on remote animals (Cary & Massecar, 2017; and Cushing, 2017).  Objective evidence, which up to now has been in short supply due to the legal limitations on remote prescribing, is needed to inform this debate, so that rational judgements can be made for the benefit of animal health and welfare based on fact rather than subjective opinion. A recent literature review on the subject (Teller & Moberly, 2020) suggests that the few published reviews of direct to consumer (DTC) telemedicine have been favourable, and that if human paediatrics is used as an analogy to veterinary medicine, studies in paediatric telehealth can lead to cautious optimism for veterinary medicine to observe similar outcomes.

### The aim of the audit was to:

- perform an initial data exploration to assess if any harm was caused and the efficacy of remote prescribing (POM-V / POM) medications via remote video telemedicine veterinary-led consultations by looking at outcomes.

### The objectives of the audit were to assess:

- the outcome of remote prescribing (POM-V / POM) in terms of complete / expected response to treatment, partial response to treatment with referral into a physical practice, no response, or adverse event; grouped into separate subcategories according to body system / disease categories;
- the prescribing of antibacterial medications.

## METHODS

A clinical audit was performed on veterinary-led video consultations via a dedicated smartphone veterinary telemedicine application (app.). The audit involved a single veterinary telemedicine company, with (at the time period of the audit) 50 RCVS registered veterinary surgeons licensed to practice in the UK, all of whom participated in video consultations remotely. The number of veterinarians fluctuated during the audit period; all veterinarians worked part-time hours on a flexible and varied rota basis.

There is no physical veterinary practice associated with the company. The audit involved review of medical records but did not involve review of consultation video recordings. This was a retrospective analysis of data from clinical records and client communications via emails stored in the company’s secure cloud storage. All records that had a remote consultation via the app. between 1 April–31 October 2020, whilst the RCVS temporary remote prescribing guidance period was active, were retrieved.

All consultations consisted of both a visual video and audio link with the client and pet. A remote consultation was only considered ‘completed’ if internet connectivity / audio and visual quality was sufficient to allow for history taking and assessment of the pet.

Descriptive details of all pets having a veterinarian video consult were retrieved and assessed pertaining to:

- Signalment (species, age, breed, gender)
- Body system / disease categories managed remotely classified into 15 sub-categories according to primary veterinary nomenclature (VeNOM) code presumptive diagnosis selected by consulting veterinarian:
- Integument
- Musculoskeletal
- Parasitic
- Ophthalmic
- Gastrointestinal
- Cardiorespiratory
- Trauma / external factor
- Urinary & renal
- Reproductive
- General / systemic / metabolic
- Dental
- Neurological
- Behavioural
- Neoplastic & miscellaneous masses[1]
- Non-specific[2]

Every consultation resulting in a POM-V / POM prescription was pulled from a MySQL relational database and tracked to resolution. MySQL is a relational database management system that is based on structured query language (SQL) (MySQL, 2022). These consultations were identified through the product ordering system, allowing for identification of the client, product purchased and date of purchase. Every 2 weeks the consultation was assessed in the company’s practice management system to ensure that the client had returned for their follow-up, and to note the outcome of the given treatment and condition.

For consults where POM-V / POM medications were prescribed the following data was collected:

Details of different prescription medication (POM-V / POM) classes prescribed:

- Oral antimicrobial
- Topical containing antimicrobial
- Analgesics (NSAIDS, paracetamol ± codeine, gabapentin, tramadol)
- Parasiticide
- Antipruritic[6]
- Other[3]

Outcomes of treatments with POM-V / POM medications were classified into one of the following mutually exclusive categories:

Complete / expected response to treatment

Partial response to treatment – (all pets referred into a physical practice)

No response to treatment*

Adverse event

No treatment outcome available (no follow-up available / prescription medication not administered)

*Sent to clinic for physical examination or for further diagnostics / procedure, or followed-up with further remote care with a change of treatment plan.

Data was aggregated and anonymised so that no person or pet could be identified from the presentation of results in this publication.

Descriptive data was then prepared and presented in graphical format to understand the clinical outcomes and inform the veterinary and wider business team. Descriptive statistics were produced using data extracted from our data warehouse (Google BigQuery, 2022) using SQL programming language (MySQL, 2022; Google BigQuery, 2022) and visualised through a combination of Microsoft Excel and Python Seaborn. All queries were saved as views enabling them to be audited and re-run as required. The data extracted for this paper included consultation random universally unique identifier (UUID), consultation date, consultation type, outcome, pet UUID, species, gender, pet age at time of consultation, presenting problem, diagnosis, product name, product UUID, product legal category, product class, product European Medicines Agency (EMA) classification, product antibacterial active ingredient, product oral / topical grouping, product antifungal / antibacterial grouping, and date product shipped.

## RESULTS

### Number of consults and prescriptions

21,383 veterinary video consults were undertaken via the app. during the study period. 78.1% (16,695) were dogs and 21.9% (4,688) were cat consults. The mean age for dogs was 4.9 years (median 3.8 years). The mean age for cats was 5.6 years (median 5 years). The male:female ratio was 10:7 for dogs and 2:1 for cats.

**Figure 1:**
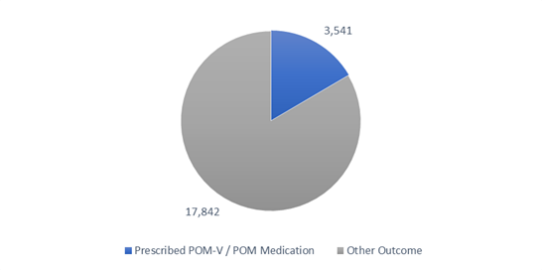
Total completed video consultations with and without POM-V / POM prescriptions for the audit period

Of the total 21,383 vet-led video consults completed in this timeframe, 16.6% (3,541) had a POM-V / POM prescribed during the consultation (Figure 1).

A pie chart depicting the completed remote veterinary led video consultations via a dedicated veterinary telemedicine smartphone app. between 1 April–31 October 2020, showing the split of consultations that resulted in remote prescribing of POM-V / POM medication, and those that had ‘other’ outcomes (follow-up consultation with remote veterinary team; recommend in person veterinarian visit [physical exam required]; alternative product recommendation [NFA-VPS, AVM-GSL]; no concern noted; monitor at home; recommend in person veterinarian visit [emergency]; laboratory testing).

The other 17,482 consultations that did not have a prescription medicine prescribed had the following individual or combination of resolutions assigned (total of 22,131 resolutions):

- Follow-up consultation with remote veterinary team: 6,421
- Recommend in person veterinarian visit (physical exam required): 4,852
- Alternative product recommendation (NFA-VPS, AVM-GSL): 4,216
- No concern noted: 3,151
- Monitor at home: 2,313
- Recommend in person veterinarian visit (emergency): 959
- Remote laboratory testing (samples collected by owners at home and analysed by external laboratory): 219

### Breakdown of Prescriptions

Of the 3,541 vet-led video consultations that had a prescription, some consultations resulted in more than one medication being prescribed.    

There were 4,282 POM-V / POM medications prescribed in total between 1 April–31 October 2020; 96.1% (4,117) were POM-V, and 3.9% (165) were POM (prescribed following the prescribing cascade). POM-schedule medications (7) have been included in the POM / POM-V totals above.^ [5]^

**Figure 2:**
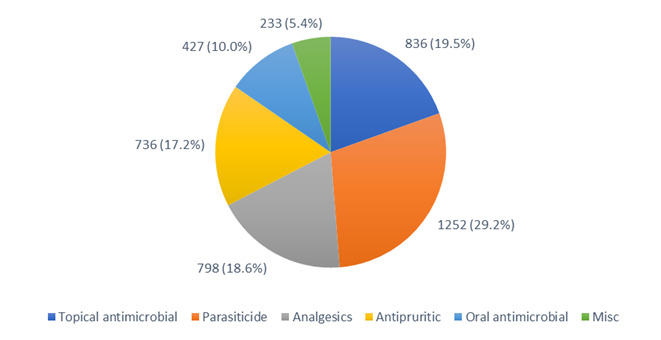
POM-V / POM medication classes prescribed remotely during the audit period

For consults where POM-V / POM medications were prescribed, prescription medications divided into the medication classes described in the methods can be seen in Figure 2.

A pie chart depicting the POM-V / POM medication classes prescribed as a result of completed remote veterinary led video consultations via a dedicated veterinary telemedicine smartphone app. between 1 April–31 October 2020, showing the grouping into broad medication classes. 4,282 POM-V / POM medications were prescribed, from 3,541 vet-led video consultations. Medication broad classifications included oral antimicrobials, topicals containing antimicrobials, analgesics, parasiticides, antipruritics and other / miscellaneous.^ [3]^

**Figure 3:**
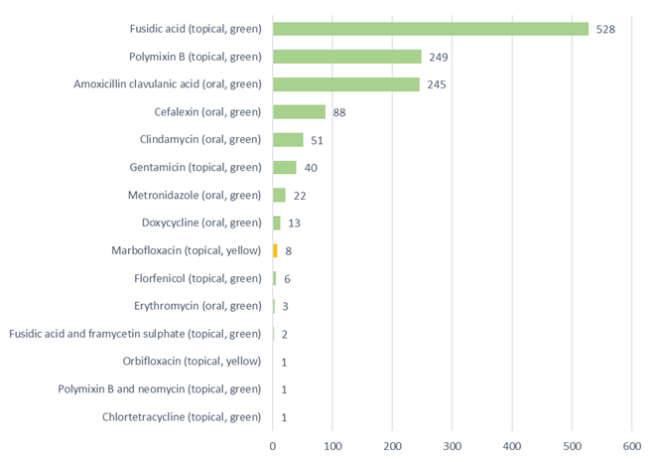
Antibacterial POM-V / POM medications prescribed remotely during the audit period

Antibacterials were classified using the traffic light (red / yellow / green) system utilised by the BSAVA / SAMSoc Protect Me Guidance as recommended by the RCVS and BSAVA (BSAVA, 2018) (Figure 3).

Of the 1,258 antibacterial prescriptions (five antimicrobial products were not included due to them being anti-fungal); 99.3% (1249) were ‘green’ first-line antibacterials, 0.7% (nine) were ‘yellow’ highest priority critically important antibacterials, all of which were topical, and no ‘red’ restricted antibacterials were prescribed. The nine yellow, critically important antibacterials were topical fluoroquinolone ear medications (eight marbofloxacin, Aurizon; one orbifloxacin, Posatex). 66.7% (six) of these were prescribed after culture and sensitivity was performed on an ear swab. 33.3% (three) were prescribed first line after no culture and sensitivity. 33.5% (422/1258) were oral antibacterials and 66.5% (836/1258) were topical antibacterials, (five oral antifungals included as antimicrobials in Figure 2 are not counted here as they are not antibacterial).

A bar chart depicting the antibacterials prescribed as a result of completed remote veterinary led video consultations via a dedicated veterinary telemedicine smartphone app. between 1 April–31 October 2020, and the application route (topical or oral). (Green = first line antibacterials, yellow = highest priority critically important antibacterials, red = antibacterials with restricted use in human medicine [none prescribed]).

### Co-prescribing of medications

**Figure 4:**
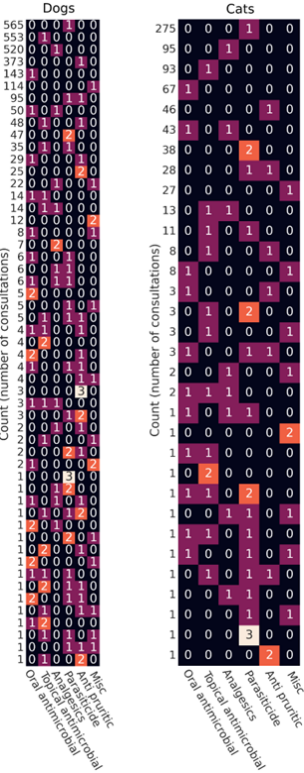
Counts of the number of consultations in which combinations of POM-V . POMs falling into product broad classification were prescribed

Co-prescriptions are best visualised across product families by a heatmap of the row signatures as displayed in Figure 4, where each row represents the number of consultations (count) in which a particular combination of medications was prescribed. The heatmaps, separated out for dogs and cats, include both POM and POM-V prescriptions, showing a large variation in prescribing patterns.

Medication broad classifications included oral antimicrobials, topicals containing antimicrobials, analgesics, parasiticides, antipruritics and other / miscellaneous.^ [3]^

Body system / disease category

**Figure 5:**
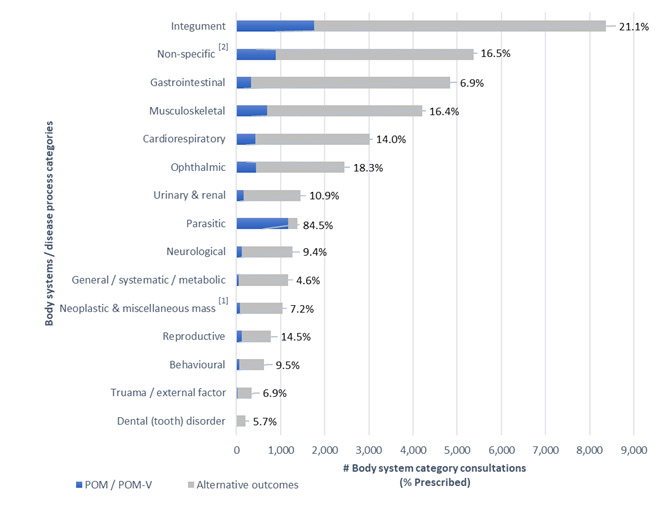
Remote consultations with and without POM-V / POM prescriptions, grouped into body system / disease category

Conditions managed remotely that had a remote prescription were classified into 15 body system / disease category presumptive diagnosis subsets according to primary veterinary nomenclature (VeNOM) code selected by consulting veterinarian (Figure 5):

- Integument
- Musculoskeletal
- Parasitic
- Ophthalmic
- Gastrointestinal
- Cardiorespiratory
- Trauma / external factor
- Urinary & renal
- Reproductive
- General / systemic / metabolic
- Dental
- Neurological
- Behavioural
- Neoplastic & miscellaneous mass^[1]^
- Non-specific^[2]^

A graph depicting all body system / disease categories managed remotely by completed remote veterinary led video consultations via a dedicated veterinary telemedicine smartphone app. between 1 April–31 October 2020; showing the number of consults grouped into 15 body system / disease category presumptive diagnosis subsets according to primary veterinary nomenclature (VeNOM) code selected by the consulting veterinarian at the time of video consultation. Blue segments show the proportion of cases for each category that were treated with a POM-V / POM via remote consultation (percent figure of prescriptions for that body system / disease category shown numerically at end of line). Grey segments had alternative outcomes that did not involve the prescription of POM-V / POM medication via remote means; veterinarians were able to allocate multiple VeNOM codes per consult to the non POM-V / POM consults.

The top five body system / disease categories appointed during all consultations between 1 April–31 October 2020 in descending order were integument, non-specific^[2]^, gastrointestinal, musculoskeletal and cardiorespiratory (Figure 5).

The top five body system / disease categories from all consultations across all body systems that were prescribed a prescription medication in descending order were integument, parasitic, non-specific^[2]^, musculoskeletal and ophthalmic (Figure 5).    

Dermatological disease (integument) was the most common body system / disease category that was seen through the app. and had the greatest volume of prescription medications.

However, when looking at the percentage of prescriptions for each specific body system consultation (Figure 5) it was second most prescribed for (21.1% = 1,765 prescriptions / 8,367 dermatology consults), with parasitic being the highest with a 84.5% prescription rate for all parasitic consultations (1166 prescriptions / 1380 parasitic consults). Non-specific^[2]^ body system / disease categories made up the second most common consultation seen and third most prescribed for during this time. The non-specific^[2]^ sub-category includes rechecks and consultations where no body system / disease category was applicable. Gastrointestinal cases were the third most common consultation seen but with a relatively lower prescribing rate (6.9% = 333 prescribed / 4,845 gastrointestinal consults).

### Outcomes of treatment following remote prescription

**Figure 6:**
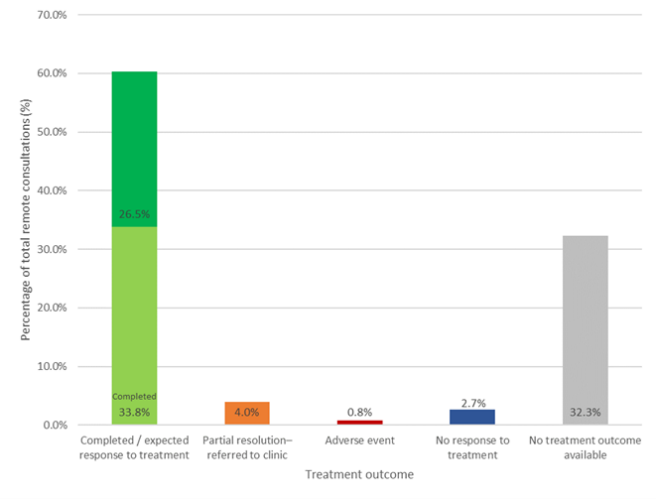
Treatment outcomes for consultations with remote prescriptions

Outcomes, based on analysis of clinical notes and/or response to client feedback email, of treatments with POM-V / POM medications were classified into one of the below categories (Figure 6):

- Complete / expected response to treatment
- Adverse event
- No response to treatment
- Partial response to treatment (with all pets referred into a physical practice)
- No treatment outcome available

A graph depicting outcomes of treatment for patients managed remotely that had a POM-V / POM prescription as a result of completed remote veterinary led video consultations via a dedicated veterinary telemedicine smartphone app. between 1 April–31 October 2020; showing the outcomes of treatments with POM-V / POM medications separated into the following outcome categories: complete / expected response to treatment; partial response to treatment with referral into a physical practice; adverse event; no response to treatment; and no treatment outcome available.

### Complete / expected response to treatment

60.3% (2,135/3,541) of remote consultations which were prescribed a POM-V / POM product resulted in the outcome complete or expected response to treatment.

- 33.8% (1,197 /3,541) complete response / resolutions of clinical signs after follow-up appointment or email
- 26.5% (938/3,541) expected response to treatment (complete resolution of signs not expected in these cases most requiring chronic ongoing medication e.g., non-steroidal anti-inflammatory drugs (NSAID) for chronic osteoarthritis, or anti-pruritic for atopic dermatitis)

Of the consults where the outcome was known (when the outcome category ‘no treatment outcome available’ was removed) 89.0% (2,135/2,399) of all treatment outcomes were complete / expected response to treatment.

### Partial resolution – referred to clinic

4.0% (140/3,541) of remote prescriptions needed to be referred to a clinic for reasons including;

- Further diagnostics (radiography, blood tests, eye examination)
- Procedures (ear flush, abscess lance)
- Injectable or topical treatments requiring veterinary administration

### No response to treatment

2.7% (94/3,541) of remote prescriptions did not respond to treatment. These patients were then either:

- Sent to clinic for physical examination
- Sent to clinic for further diagnostics / procedure
- Followed-up with further remote care with a change of treatment plan

### No treatment outcome available

Of the 3,541 consultations in which a prescription was purchased, 32.3% (1,142/3,541), had no treatment outcome available for the following reasons:

- 30.4% (1,078/3,541) No feedback received (lost to follow-up despite multiple attempts)
- 1.8% (64/3,541) patients were not administered prescribed medication for reasons including:
  - postal delay / out of stock (those cases were referred into practice)
  - condition deteriorated prior to commencement of treatment and were referred into practice
  - symptoms resolved before medication was administered
  - order cancelled by owner

### Adverse events

Assessment for possible adverse events was undertaken at follow-up examination or by follow-up email (if a second consult was not attended).

There were 30 adverse events (0.8%, 30/3,541) identified following POM-V / POM medication remote prescription, from use of 11 suspected products (with three further products used concomitantly) within the reporting period. Overall, the signs seen were mild and predominantly associated with the gastrointestinal tract, 29/30 reactions involved vomiting or diarrhoea. All adverse events were reported to the VMD.

From our body system / disease categories groupings 33.3% (10/30) were prescribed for the musculoskeletal system, 30% (9/30) for parasitic disease, 26.7% (8/30) for the integument system, 6.7% (2/30) for gastrointestinal disease and 3.3% (1/30) for cardiorespiratory disease.

From our prescription medication groupings, 43.3% (13/30) of adverse events were seen after NSAIDs, 30.0% (9/30) after an anti-parasitic product, 10.0% (3/30) after an oral antimicrobial, 10% (3/30) after an antipruritic, and 6.7% (2/30) after oral steroids^[4]^.

6.7% (2/30) were sent into practice for examination and treatment. 93.3% (28/30) were confirmed resolved during follow-up consultation or email. We were unable to attain follow-up information for two patients (6.7%).

Over half (56.7%, 17/30) the adverse events reported were identified by follow-up email for people that had failed to attend a follow-up consultation. If this process had not been followed, we may not have been informed of these mild adverse events.

### Further breakdown of prescribing outcomes and medication classes

**Figure 7:**
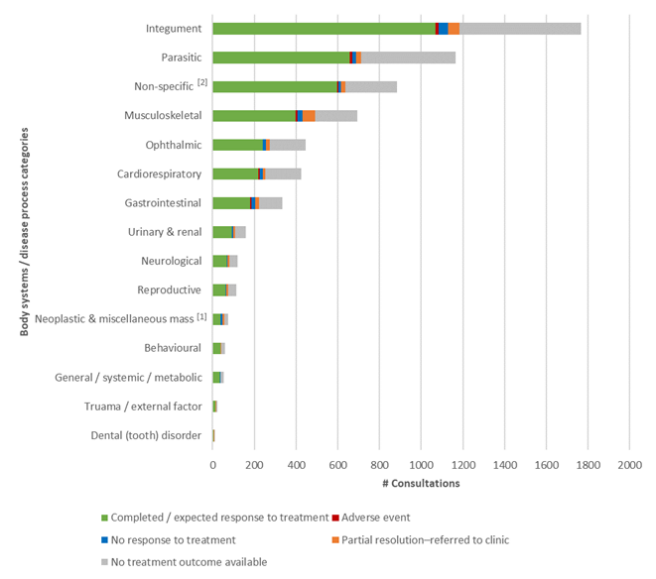
Patient outcomes for consultations with remote POM-V / POM prescriptions for the audit period grouped by body system / disease categories

**Figure 8:**
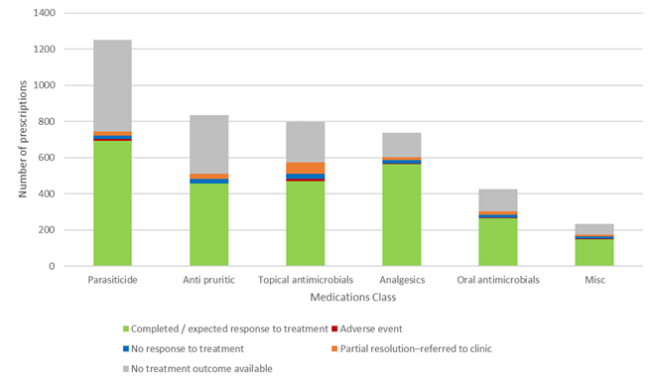
Patient outcomes for remote POM-V / POM prescriptions for the audit period grouped by medication class

Outcomes of consultations where medication was prescribed divided into body system / disease category (Figure 7) and patient outcomes per prescription medication class (Figure 8) show that the majority of the prescribing outcomes in every category was expected / complete response to treatment, with a fairly even distribution across the sub-categories.

A graph depicting outcomes of treatment for veterinary patients (cats and dogs) managed remotely that had a POM-V / POM prescription as a result of completed remote veterinary led video consultations via a dedicated veterinary telemedicine smartphone app. between 1 April–31 October 2020; grouped into 15 body system / disease category presumptive diagnosis subsets according to primary veterinary nomenclature (VeNOM) code selected by the consulting veterinarian at the time of video consultation. The outcomes of consultations with POM-V / POM medications separated into the following outcome categories: Complete / expected response to treatment; adverse event, no response to treatment; partial response to treatment with referral into a physical practice; and no treatment outcome available.

A graph depicting outcomes of treatment for veterinary patients (cats and dogs) managed remotely that had a POM-V / POM prescription as a result of completed remote veterinary led video consultations via a dedicated veterinary telemedicine smartphone app. between 1 April–31 October 2020. Outcomes grouped by the following prescription medication class groups: oral antimicrobial; topical containing antimicrobial; NSAIDs and paracetamol with codeine; parasiticide; antipruritic^[6]^; and other^[3]^.

**Figure 9:**
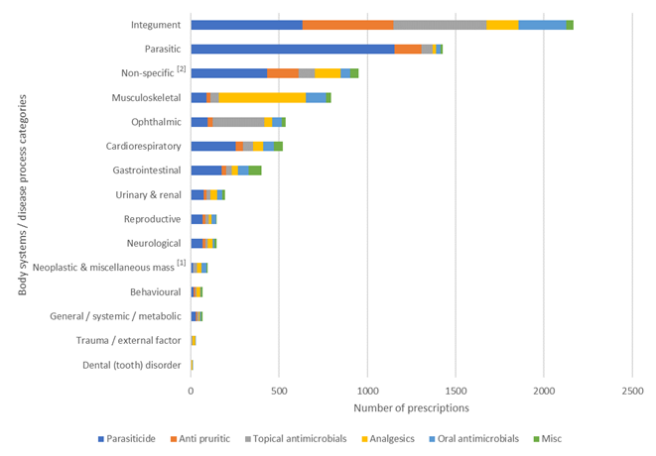
Medication class subsets for remote POM-V / POM prescriptions for the audit period grouped by body system / disease categories

The number of prescriptions issued for each body system / disease category broken down into medication classes prescribed is shown in Figure 9. The majority of prescriptions issued for the:

- Integument body system / disease categories were distributed between the anti-pruritic, topical antimicrobial and parasiticide medication classes
- Musculoskeletal body system / disease category were for the nonsteroidal / paracetamol medication class
- Parasitic body system / disease category were for the parasiticide medication class
- Ophthalmic body system / disease category were for the topical antimicrobial medication class
- Non-specific body system / disease category were for the parasiticide medication class

A graph depicting different classes of POM-V / POM medication prescribed (oral antimicrobial; topical containing antimicrobial; NSAIDs and paracetamol with codeine; parasiticide; antipruritic (oclacitinib, anti-histamines, topical hydrocortisone spray, topical steroids without antimicrobial, oral steroids); miscellaneous^[3]^) grouped into 15 body system / disease category presumptive diagnosis subsets according to primary veterinary nomenclature (VeNOM) code selected by the consulting veterinarian at the time of video consultation, as a result of completed remote veterinary led video consultations via a dedicated veterinary telemedicine smartphone app. between 1 April–31 October 2020.

## DISCUSSION

### Big picture: What gaps does our research fill? Why is this important?

Peer-reviewed evidence examining whether remote prescribing by a dedicated veterinary telemedicine route can be efficacious and safe for patients, is currently not available in the veterinary literature. A recently published comprehensive review of veterinary telemedicine concluded that most information regarding direct-to-consumer telemedicine, although favourable, was gleaned from human healthcare, and therefore more relevant evidence was needed from the veterinary sector (Teller & Moberly, 2020). Our hope with this audit is to begin the process of presenting real data, so that the RCVS and other legislative bodies will be able to develop best practice for the future of veterinary telemedicine based on evidence. Looking to the human medical sector, where telemedicine is a topic of significant discussion, teleconsultation appears to work well for both acute and chronic disease management (Burke et al., 2015). Shigekawa et al. (2018) deemed human telehealth to be equivalent to in-person care. Another study in paediatrics, which are deemed most close to animals due to restricted communication, suggested that the assessment of febrile children by remote assessment was as reliable as bedside assessment (Siew et al., 2016). McSwain et al. (2017) suggested that children under 2 years of age are not appropriate candidates for telemedicine, due to the higher risk nature of children of that age, and not due to their lack of ability to communicate. Therefore, extension of this statement to animals on the basis of their lack of communication is a false conclusion to draw (Teller & Moberly, 2020).

Remote prescribing, as part of direct-to-consumer telemedicine provision, may prove valuable to clients, pets and veterinarians in certain situations for reasons including earlier intervention, reduced stress and anxiety both for patient and client, enhanced continuum of care, clinically vulnerable people, accessibility, affordability, convenience, time management, and safety of the veterinarian. Pet owners have indicated that they would prefer to be able to continue to obtain POM-V / POM medicines via remote teleconsultation (YouGov, 2021). The current RCVS definition of ‘under your care’ (currently under review) which governs the right to prescribe and limits it to in-person physical examination, is ambiguous and there is disparity between prescribing for a herd of farmed animals and individual pets. It does not currently allow for remote prescription to pets without a physical in-person examination. We hope that the data provided in this audit will help to guide the RCVS (and other professional bodies) in their decision-making regarding future governance of remote prescribing, that there are certain situations, in well-structured and governed telemedicine, where remote prescribing does not cause harm and may even be of benefit to animal health and welfare.

### Critical analysis of the major findings, additional findings and literature comparison

Assessment of the data gathered during our audit prescribing period has shown the following:

#### A low prescribing rate per number of video consults

Prescription medications were only issued in 16.6 out of every 100 app. based veterinary video consultations. A third of the consultations that did not result in a prescription being issued were referred into practice or had further laboratory testing, whilst the other two thirds were either recommended a non POM-V / POM product, were monitored, had a follow-up veterinary telemedicine appointment or were not of concern.

There is limited data to show the percentages of in-practice consultations that lead to a POM-V / POM prescription in small animal general practice in the UK. A VetCompass study (Elkholly et al., 2019) of primary care records of UK dogs found a systemic glucocorticoid prescribing rate of 6.2% (28,472/455,557 dogs in a 1 year period). A second publication examining systemic glucocorticoid prescriptions in three UK veterinary practices found a systemic glucocorticoid prescribing rate of 15.3% (4790/31,273) of dog and cat consults, but with a great deal of inter-practice variation in prescribing patterns (O’Neill, 2012). Our systemic (oral) glucocorticoid prescribing rate was 0.8% (177/21,383). However, this may in part reflect the disparity between telemedicine vs in practice case loads, and the fact that no injectable medications were administered by nature of the remote means of consultation. Ours is also a smaller case load, from just one ‘practice’ and over 7 months rather than a year’s duration.

Therefore, it is currently impossible to compare prescribing rates directly between a dedicated telemedicine provider and a general practice. We would welcome an audit of a general practice’s prescribing rates to see how these compare. We suspect that there is a critical difference in the case presentation population for a smartphone veterinary telemedicine app. when compared to that of general practice, and so granularity of data on case types would need to be available for a robust comparison.

Some prescribing data from small animal practice has been published that can be used for comparison. A Small Animal Veterinary Surveillance Network (SAVSNET) study by Singleton et al. (2018), examined medical records from 457 companion animal practices over a 2 year period for prescribed pharmaceutical agents, finding 65% of dog and 69% of cat consultations had a prescription. One major difference with that study was that it included vaccines, which we did not administer by telemedicine. They found a POM (human-only use authorised medicine) prescribing rate of 5.1% of all prescriptions in dogs and 2.1% for cats. Our POM prescribing rate was comparable to these figures at 3.9% (165/4,282). They used a novel mapping method to examine differences in prescribing patterns by species, based on presenting complaint, as well as looking for groups of drugs commonly co-prescribed which is something that we could further do with our data. We examined co-prescription and found a large variation, but in the future, we could also examine the differences more closely by examining presenting complaints along with this. We have not generally separated our data in cats and dogs as the numbers would have been smaller and our aim was to gather an overall picture in general for this audit.

Summers et al. (2014) found a prescribing rate of 97% (659/683) for antimicrobials alone in 683 presenting cases of canine pyoderma out of 54,600 dogs presented to 73 participating practices in the UK in 2010. Of all dermatological cases presenting in the audit period, only 21.1% (1,765/8,367) received any form of prescription treatment.

Singleton et al. (2019) found the most common prescription medication provided to a cohort of canine acute diarrhoea cases to be systemic antimicrobials, given to 49.7% of cases in their study population of 3,189 cases (3,159 dogs) collected from 179 volunteer veterinary practices between April 2014 and January 2017. This compares with 6.9% (333/4,845) of gastrointestinal cases presenting during the audit period being prescribed any POM / POM-V medication in our app. As previously suggested, it may be that the reason for our apparent difference in prescribing patterns is that our case population differs from that seen in general practice, in that we see cases earlier and we see cases that otherwise would not have been presented for veterinary examination.

The relative proportions of consultations resulting in POM-V / POM prescriptions vs those that did not for the different subsets of body system / disease categories managed remotely cannot be easily compared across categories because they are likely to be influenced by a category-dependent selection bias. This is simply because certain disease categories are more amenable to remote assessment. This is borne out in the numbers and appeals to common sense: skin conditions are more easily assessed because the clinician can see the affected area clearly and perform a satisfactory clinical examination, despite being remote from the patient. As for the absolute proportions, without data from an in-clinic setting, there is no distribution on which to base a null hypothesis for a standard hypothesis test.

#### Categories of types of cases seen

Although not the main aim of this audit, it is worth briefly considering the broad types of cases seen through our app., that were categorised into body system / disease category subsets to gain insight into the types of cases in a remote video consultation practice. The largest caseload, which also had the largest number of prescriptions across all body systems, was dermatological cases. The second most common consultation reason category was ‘non-specific’ cases, including consultations where no body system / disease category was applicable, 45% (432/951) of which were for parasite prophylaxis. Gastrointestinal cases were the third most common consultation seen yet prescribing for gastrointestinal cases was low. Musculoskeletal, cardiorespiratory and ocular cases were the next three most common categories.

A paper presenting the common presenting conditions in UK small animal practice found skin to be the most commonly affected body system, followed by non-specific problems, then gastrointestinal problems (Robinson et al., 2015). This is similar to our caseload for the most common body system / disease category, however the case presentations in the less frequent categories differed slightly, with a notable higher prevalence of dental, respiratory, cardiovascular and renal cases compared to our data. They also examined low numbers (2,158 dogs and 881 cats), so data from a larger dataset from physical companion practice would be an insightful comparison. We plan to further thoroughly examine this data in detail with our growing dataset and publish our findings as a subsequent audit, to aid better understanding of case types managed by our telemedicine app.

#### Antibacterial stewardship

The majority of literature on prescribing rates in general practice relate to antibacterial use. It is a major concern within the profession that prescription via teleconsulting will result in overuse of antibacterials and fuel a selection bias for increased antibacterial resistance (Magalhães-Sant'Ana et al., 2020). This concern is valid considering the evidence that Ray et al. (2019) produced showing increased antimicrobial prescribing rates in human telemedicine via remote means.

Some multicentre studies have examined antibacterial prescribing rates in UK companion animal practice. A SAVSNET study by Singleton et al. (2017) recorded an antibacterial prescription was dispensed for 18.8% of dogs and 17.5% of cats seen in first opinion practices across England and Wales over a 2 year period (2014–2016) from 457 veterinary practices. A VetCompass 2 year study of 374 UK companion animal practices revealed an antibacterial prescribing rate of 25% (242,736/963,463) in dogs and 21% (122,594/594,812) in cats receiving at least one antibacterial, with 42% of those receiving a further antibacterial (Buckland et al., 2016). Radford et al. (2011) recorded rates as high as 35.1% (5,519/15,727) in dogs and 48.5% (2,708/5,587) in cats for UK pets, assessing data over a 3 month period from 16 small animal practices in England and Wales.

Our data showed that during the audit period, antibacterials were prescribed in 5.9% (1,258/21,383) of all veterinary consultations (2.0% (422/21,383) oral preparation / 3.9% (836/21,383) topical containing antibacterial). The majority of the topical antibacterials prescribed were for dermatological cases and ophthalmological cases. The majority of the oral antibiotics were prescribed for dermatological cases (predominantly allergic skin disease) and trauma cases (bites, burns or infected wounds). There were low numbers of urinary or respiratory case numbers, which in clinical practice may be expected to result in a higher rate of oral antibacterial usage. Deeper understanding of any differences in case loads between remote consultations vs a traditional veterinary setting would make an interesting and useful comparison.

Our antibacterial prescribing rate is significantly lower than the prescription rates in physical UK first opinion small animal practice. Again, the apparently lower rates of antibacterial prescribing we are reporting could be a reflection of a different caseload, or due to increased clinician vigilance on remote prescribing of antibacterials, or a combination of both. Prescribing patterns have changed over the recent years with the increasing awareness of antibacterial prescribing best practice and antimicrobial resistance. As use of antimicrobials is a key driver for development of antimicrobial resistance, it has been highlighted as vital that the veterinary profession embraces the responsible use of antimicrobials, to safeguard human and animal health, and to preserve the right to prescribe certain antimicrobials that are important in human medicine (Singleton et al., 2021). Companion animal veterinary practitioners have been shown to respond to involvement in structured antimicrobial stewardship programmes (Singleton et al., 2021).

During the audit period we prescribed 1,258 antibacterials. The Joii antibiotic prescribing policy utilises the traffic light (red / yellow / green) system based on the BSAVA / SAMSoc Protect Me Guidance as recommended by the RCVS and BSAVA (BSAVA, 2018) and additional support from internal medicine specialists at the Royal Veterinary College.

Of the 1,258 antibacterial prescriptions; 99.3% (1,249/1,258) were green first-line antibacterials, 0.7% (9/1,258) were yellow highest priority critically important antibacterials, all of which were topical, and none were red restricted antibacterials. 33.5% (422/1,258) were oral antibacterials and 66.5% (836/1,258) were topical antibacterials. BSAVA / SAMSoc guidance advises that ‘topical preparations reduce selection pressure on residential intestinal flora (microbiome)’ so this route was utilised preferentially when it was clinically indicated.

As a result of this audit, our antibiotic prescribing guidelines will ensure to highlight the importance that first line topical ear preparations are prescribed first, ideally after culture and sensitivity, and critically important antibacterials are reserved for those cases which have not responded to first line treatment or have been shown to require these antibacterial classes on culture and sensitivity.

The European Medicines Agency (EMA) classifies antibacterials into four categories; A (red) avoid, B (orange) restrict, C (yellow) caution, and D (grey) prudence; slightly different to the traffic light system of the BSAVA / SAMSoc Protect Me guidance that we employed. The categorisation of polymixin B by the EMA (EMA website) as a category B restricted antibacterial is different to the BSAVA / SAMSoc Protect Me guidance, where polymixin B topical ear medication is a recommended first line treatment for otitis externa. During the audit period polymixin B (Surolan) was our first line topical ear medication following the guidance and also taking into account there was a stock issue with fusidic acid and framycetin sulphate (Canaural). The classification of polymyxin B is something we will monitor within the UK guidance and alter our antibacterial prescribing protocols accordingly. As it has been classified as restricted in Europe we will guide our veterinarians to utilise other first line topical ear medications as a priority.

#### Efficacy of treatment

Complete and expected response to treatment accounted for 89% (2,135/2,399) of consultations where prescription medication was prescribed where follow-up information was available (60.3% (2,135/3,541) of all consultations where prescription medication was prescribed, if those where no treatment outcome was available are included).

A low rate (2.7%, 94/3,541) of consultations where prescription medication was prescribed had shown the medication was not effective in treating the patient. The majority of the remainder were lost to follow-up and a small proportion had a partial response but were referred into practice.

There was no pattern in terms of condition type that resulted in poor remote outcome after prescribing. In terms of medication type, prescribed pain relief appears to have marginally higher figures of partial response to treatment than other medication types. Further analysis of the cases would be needed to determine the exact cause of this. We suspect that, due to the nature of remote prescribing during COVID-19, that a significant number of these cases were prescribed NSAIDs purely as a temporary measure until the patient was able to be seen at their local veterinary practice and assessed for further diagnostic tests or medication.

#### Follow-up engagement was high

Follow-up was achieved for the majority of prescription medications issued in this audit. We were unable to attain follow-up in 32.3% (1,142/3,541) of cases despite multiple attempts to contact owners. Therefore, we are blinded to the outcome of those cases and it is not possible to infer or extrapolate what may have happened with those cases – this is a limitation of this study. There is no comparison available in the literature to show how this measures up to general practice. We were proactive in encouraging follow-up for POM-V / POM prescribed cases as we are aware that remote prescribing is unchartered territory and we wanted to ensure a high clinical standard of care for our patients. Follow-up via a video link, or failing that an email exchange with owners, may be a less frictional way of attaining follow-up data than requesting re-examination at a physical practice. Therefore, it is probable that we have a higher follow-up attainment compared to clinical practice, though this is purely speculative.

#### No harm caused

We had a low rate (0.8%, 30/3,541) of adverse reactions to remotely prescribed POM-V / POM medications reported, which was an adverse event rate of 0.14% (30/21,383) from total remote consultations during the audit period. All adverse reactions were reported to the veterinary medicines directorate (VMD). All were mild in severity, in most cases vomiting or diarrhoea that fully resolved[4].

Our data shows 30.0% (9/30) adverse events occurred after parasiticides and 43.3% (13/30) occurred after anti-inflammatories. These medication groups were in the top five groups accountable for all adverse events in all animals reported to the VMD in their latest pharmacovigilance summary report (VMD, 2019).

The VMD online dashboard (VMD, 2019; VMD, 2020) shows that 14.6% (6,369/43,619) of all clinical signs reported with adverse events in cats and dogs in the UK were due to anti-parasiticides and 6.7% (2,910/43,619) due to anti-inflammatories. Gastrointestinal signs were commonly seen and from all reports submitted to the VMD in 2019, emesis (1st for anti-inflammatories, 2nd for anti-parasitics) and diarrhoea (4th for anti-inflammatories, 7th for anti-parasiticides) ranked within the top ten signs reported for both classes of medication. The clinical signs and medications we have reported here fit with those commonly reported to the VMD.

A VetCompass study examining side effects of glucocorticoids reported within 31 days of treatment, over a 1 year prescribing period, found 4.9% of patients to have side effects of polyuria, polydipsia, diarrhoea and or vomiting following glucocorticoid administration (Elkholly et al., 2020).  The risk of side effects was increased with use of oral glucocorticoids either alone or in combination with injectable, compared to use of injectable alone. Our own oral glucocorticoid prescribing rate was low, with a lower adverse event rate (1.1%) than that reported in the VetCompass study. We did not have reports of polyuria or polydipsia as adverse events in this audit. Our follow-up times were in most circumstances much sooner than 31 days (typically within 1–4 days of the end of a treatment course), therefore we could have missed some patients developing these signs. Thus highlighting that we need to change company policy to include asking questions around these clinical presentations and also to pursue longer-term adverse event and side effect reporting on follow-up of remote prescribing cases.

It is hard to find comparable figures for adverse events from general veterinary practice. Evidence relating to oral and topical medications is not readily available, most likely due to the under-reporting of adverse events to the VMD.

The VMD are currently funding a PhD project and a masters project in collaboration with SAVSNET (Small Animal Veterinary Surveillance Network (SAVSNET) – University of Liverpool, 2021) which may in future help answer questions on how many adverse events get reported compared to the number that occur in practice.

We were proactive in enquiring about adverse reactions to every client where a POM-V / POM had been issued. Over half (56.6%, 17/30) the adverse events reported were identified by follow-up email for people that had failed to attend a follow-up consultation. If this process had not been followed, we may not have been informed of these mild adverse events. It is probable that in physical veterinary practices, owners do not always inform the practice about a mild gastrointestinal upset after a medication has been given. Adverse events in the veterinary and human world are under reported due to many reasons including:

- Level of awareness of reporting obligations
- Length of time the product has been on the market
- increased reporting rate in the first 2 years
- Reporting environment
- social media publicity / news coverage

De Briyne et al. (2019) conducted a survey of 3,545 veterinary surgeons to gain a better insight into the adverse event reporting habits of veterinary practitioners. The findings indicated marked under-reporting. In order to increase spontaneous reporting, there is a need to make reporting easier and to make veterinarians better aware of the importance of reporting and the added value it may bring.

It is estimated that less than 10% of serious adverse events and 2–4% of non-serious adverse events are reported (Personal Communication, Veterinary Pharmacovigilance Course, 2014, Management Forum). Since we discovered over half of our adverse events, albeit a low number and mild, by proactive owner engagement following prescription, it would seem sensible that this approach be adopted and encouraged by the wider veterinary profession to ensure high standards of pharmacovigilance.

The relative proportion of outcomes of POM-V / POM remote prescription grouped by body system / disease category subsets, and for medication class groups is subject to selection bias in a similar way to prescribing vs not prescribing. The bias in this instance results from the different medications administered across the various categories. It is therefore impossible to extract any statistically significant differences between the proportions of adverse / non-adverse outcomes that is attributable solely to the remoteness of the clinician. The absolute proportions of adverse / non-adverse outcomes within each category are also difficult to assess quantitatively without in-clinic data for a null hypothesis distribution. However, it is not unlikely that adverse events are equally likely to result from data derived from in-clinic consultations, because, critically, our clinicians used their discretion in each case and used a level of caution commensurate with conducting an assessment remotely.

### Limitations of our data

#### What we are currently unable to demonstrate? Case sample and comparison to general practice

We can say that our prescribing rate seemed to be low across different body systems / disease categories, that our reported adverse events were mild and very low, that our use of antibacterials was conservative and more frequently topical than systemic. We can say that remote prescribing, in our hands, does not appear to cause harm, but this is not the same as concluding that remote prescribing is safe, per se. There were cases that were directed into practice for further examination and diagnostics, and it would likely have been unsafe to prescribe to those cases. What we cannot say is how any of this data compares to physical veterinary practices. We also have not presented a more in-depth breakdown of disease types and presentations, since that was not the focus of this audit; however, it is clear that that information is also needed to help to inform the profession on the types of cases typically encountered by a dedicated veterinary telemedicine app.

It would have been useful to analyse information pertaining to owner demographics and characteristics in order to investigate influences on outcome of therapy. However, given the retrospective nature of this study and our ethical consideration of GDPR and sensitive data regarding people, we unfortunately lack some of this information and cannot include it in this manuscript.

It is likely that clients turn to alternative options (friends / the internet / paraprofessionals) before seeking care at their local practice. What we may be seeing here is the opportunity, via a dedicated veterinary smartphone app., to intervene earlier in the process of a given disease, and therefore significantly and positively impact animal health and welfare.

#### Data set limitations

The limitations to our dataset is that it was a sample from less than a year of in-app. consultations, and during that year our caseload was growing, so may not be reflective of a typical year on year in an established online veterinary practice. We were limited to this time by the limited time period of remote prescribing relaxed rules set out by the RCVS. The audit time was also undertaken during the occurrence of a global pandemic, and so people’s behaviours and indeed prescribing decisions may have been altered due to the nature of the extenuating circumstances of a lockdown. It would be ideal to perform this audit annually on an ongoing basis, but the right to prescribe remotely would need to be approved by the RCVS in order for us to gather and then present this data.

Study design and data capture was aided by our practice management system, allowing us to collect multiple comparative data points for every case seen remotely. We have a structured clinical notes section with drop down selections and limited free text written records, to streamline our data, make it easier to explore our data, and ultimately to reduce human error. The need for any prescription medicine sale to pass through our sales system means there is no error in terms of missing cases for the audit.

Clinical staff were informed of data gathering but it was not at that time used to directly monitor performance so there should be little room for conflict of interest, although this is always possible in an audit setting.

It should be borne in mind that this review was on data from a dedicated smartphone app, where a secure video and audio link was essential for a consultation to happen, and therefore no conclusions can be drawn about remote prescribing by other means, such as email and photo exchange, or telephone conversations.

### Future directions

At this time, we have ceased remote prescribing in line with the RCVS’s temporary guidance. This means that we are unable to complete the cyclical nature of a true clinical audit, and cannot implement any learnings to better inform our prescribing protocols. If we did begin prescribing again, then stricter policy re provision of NSAIDs would need implementation, since we saw some adverse reactions predominantly to those. We would also review antibacterial prescribing and set evidence-based guidelines (where evidence available) for best practice to reduce use and use alternatives.

We would also continue to record treatment outcomes in our clinical records, and proactively engage owners for follow-up after POM-V / POM prescribing, both immediately and at longer time points following prescription, reporting adverse events to the VMD.

To really make use of our data set, we need comparative studies from other practices, both remote and in person.

### Overall conclusion and impact: take home message

In summary, through this audit we have found that remotely prescribing via our dedicated smartphone veterinary telemedicine app, we had an overall low prescribing rate, including a low antibacterial prescribing rate; our treatments were on the whole efficacious and no harm was done by prescribing remotely. We have a low rate of systemic (oral) glucocorticoid prescribing compared to physical practice. We are currently unable to compare much of our findings directly with clinical practice, due to a lack of published evidence. It is likely that the virtual practice case population is different to a physical clinic population, which could in part explain the favourable results for lack of harm by remote prescribing in our hands. More data is needed examining the case presentation types for both remote consults and physical practices, for direct comparisons to be made.

We can also conclude that proactive follow-up, both immediately during / at the end of treatment and longer term, to find out about adverse events needs to be encouraged and maintained both within our virtual practice and, we suggest, across the profession.

We invite the RCVS and other veterinary governing bodies to consider that there is a place for prescribing remotely for certain cases under certain conditions, such as a secure video and audio link via a dedicated app with clinical record keeping and pharmacovigilance practice, and that a blanket ban is not appropriate with the advance of modern technology.

The future of veterinary medicine should include remote consultations with remote prescribing where appropriate, to be able to reach as many owners and provide professional advice and appropriate treatment on pet health so that those animals that do not necessarily currently receive veterinary care do so, and to bridge the gap wanted by clients.

Footnotes[1]

Includes – lump / swelling, polyp – rectal, nasal (nose) disorder (unspecified), wart, skin (cutaneous) cyst, granuloma (site unspecified), mass lesion – skin (cutaneous), mass lesion – anal gland / sac, mass lesion – conjunctival, mass lesion – ear (aural), mass lesion – eyelid, mass lesion – nail bed, mass lesion – nose (nasal), nasal cavity, mass lesion – perianal, mass lesion (site unspecified), haematoma – other, abscess (site unspecified), congenital disorder mass lesion – vaginal, mass / swelling – skin, papilloma, solitary.

[2] Non-specific conditions include the following assigned VeNom codes – no diagnosis, not presented for problem investigation, diagnosis unchanged from last visit, not presented for a complaint, problem not identified, re-examination appointment.

[3] Miscellaneous / other medication includes the following – propentofylline, dexmedetomidine 0.1 mg/ml oromucosal gel for dogs, estriol tablets, chlorhexidine gluconate miconazole nitrate shampoo, cimetidine, bromhexine, pimobendan, frusemide ,omeprazole, selegiline hydrochloride, maropitant, phenylpropanolamine hydrochloride, cyclosporine a ophthalmic ointment, fluoxetine capsules, silver sulfadiazine cream, 1% w/v prednisolone acetate eye drops, lactulose, famotidine, ranitidine, cabergoline oral solution, carbimazole tablets, hypromellose Ph.Eur. 0.5% w/v. drops, levothyroxine sodium, thiamazole.

[4] Six cats had adverse events to prescribed medications. One cat reacted to sarolaner and selamectin (Stronghold Plus, Zoetis) in combination with emodepside and praziquantel (Profender, Vetoquinol) showing signs of vomiting and lethargy. Two cats reacted to meloxicam (Metacam, Boehringer Ingelheim) showing vomiting and diarrhoea respectively. Three cats also developed diarrhoea after medication with clindamycin (Antirobe, Zoetis), ciclosporin A (Atopica, Elanco) and amoxicillin and clavulanic acid (Synulox, Zoetis) respectively.

Of the six reactions, five resolved fully and for one, we tried but were unable to attain follow-up information.

Twenty-four dogs had adverse events to prescribed medications. Seven reactions were seen after a meloxicam product (five with Metacam, [Boehringer Ingelheim] of which one had Amoxicillin and Clavulanic acid, [Synulox Zoetis], concomitantly. Two with Meloxidyl, [Ceva]). Of these seven, two showed diarrhoea, two showed vomiting and two showed both vomiting and diarrhoea. One showed inappetance after an overdose.

Five showed reactions to moxidectin, pyrantel and sarolaner (Simparica Trio, Zoetis), one with oclacitinib (Apoquel – Zoetis, concomitant). Three vomited, one had diarrhoea and one had an upset stomach.

Four showed reactions to robenacoxib (Onsior, Elanco) three vomited and one had diarrhoea.

Prednisolone (Prednicare, Animalcare – 1, Prednidale, Dechra – 1) caused diarrhoea and lethargy respectively in two dogs. Omeprazole caused vomiting and vomiting and diarrhoea in two dogs. Diarrhoea was seen in a further four dogs, two after imidacloprid and moxidectin (Advocate, Bayer) one after spinosad A/D 85:15 (Comfortis, Elanco) and one after amoxicillin and clavulanic acid (Synulox, Zoetis).

Of the 24 reactions, 23 resolved fully and for one we tried but were unable to attain follow-up information.

[5] POM-schedule (7) were included in the POM / POM-V totals:

Pardale-V (schedule five POM-V), 

Tralieve (schedule three POM-V),

Diazepam rectal solution (POM human schedule four),

Gabapentin (POM human, schedule three).

[6] Antipruritic medications 736 (oclacitinib 391, antihistamines 75, topical hydrocortisone 67, cyclosporine 11, oral steroids 178, monoclonal antibody 10, topical prednisolone 1, immunotherapy 3). 

## Conflict of Interest

The authors declare no conflict of interest.

## Disclaimer

Any opinions expressed in articles and other publication types published in Veterinary Evidence are the author's own and do not necessarily reflect the view of the RCVS Knowledge. Veterinary Evidence is a resource to help inform, and the content herein should not override the responsibility of the practitioner. Practitioners should also consider factors such as individual clinical expertise and judgement along with patient’s circumstances and owners’ values. Authors are responsible for the accuracy of the content. While the Editor and Publisher believe that all content herein are in accord with current recommendations and practice at the time of publication, they accept no legal responsibility for any errors or omissions, and make no warranty, express or implied, with respect to material contained within. For further information please refer to our Terms of Use.
